# Supplementary material for: Variation in human herpesvirus 6B telomeric integration, excision, and transmission between tissues and individuals
Source: eLife. 2021 Sep 21;10:e70452. doi: 10.7554/eLife.70452 (PMC8492063; doi:10.7554/eLife.70452)
Supplement: Supplementary file 2. [file elife-70452-supp2.docx]

**Supplementary File 2.** Estimated Time to Most Recent Common Ancestor (TMRCA) for carriers of iciHHV-6A and iciHHV-6B with different chromosomal locations.

| **Species** | **Chromosome location** | **No. genomes** | **TMRCA ± SD** |
| --- | --- | --- | --- |
| HHV-6A | 17p | 14 | 105 033 ± 19 139 |
| HHV-6A | 18q | 5 | 22 976 ± 10 275 |
| HHV-6A | 19q | 8 | 68 928 ± 14 070 |
| HHV-6B | 9q | 11 | 20 855 ± 12 552 |
| HHV-6B | 17p (major) | 7 | 23 409 ± 11 226 |
| HHV-6B | 19q | 6 | 24 579 ± 11 260 |
