# Supplementary material for: Variation in human herpesvirus 6B telomeric integration, excision, and transmission between tissues and individuals
Source: eLife. 2021 Sep 21;10:e70452. doi: 10.7554/eLife.70452 (PMC8492063; doi:10.7554/eLife.70452)
Supplement: Supplementary file 3. [file elife-70452-supp3.docx]

**Supplementary File 3**. Distinctive features of DRR-pvT1 repeat patterns associated with various iciHHV-6B phylogenetic clades.

| **iciHHV-6B integration group** | **Distinctive features of DR_R_-pvT1 repeat patterns** |
| --- | --- |
| Provisional 2p  Example sample: 401027 | Short proximal section lacking the brown, cyan and lime green repeats ((CTAGGG), (TTAGTG) and (TTATGG) respectively) found in other iciHHV-6B proximal regions. |
| 9q  Example sample: BAN519 | Constant DR_R_-pvT1 proximal section also shared with other samples; distinctive pink, cyan, pink motif (CTATGG-TTAGTG-CTATGG) and a rare peach (TTAGAG) repeat in central section. |
| 11p  Example sample: LEIALD | Provisional distinctive feature: Absence of pink repeat (CTATGG) before the brown, green, pink, cyan, pink motif (CTAGGG-TTAGGG-CTATGG-TTAGTG-CTATGG) in the proximal region.  N.B only three samples with an 11p integration site (verified by FISH) were available for pvT1 analysis. Characterisation of pvT1 repeat patterns from other samples in the 11p-iciHHV-6B clade is needed to define the distinctive pvT1 features for this clade. |
| Provisional 17p (minor)  Example sample: 704021 | Constant DR_R_-pvT1 proximal section shared with other samples; Two dark green repeats ((TTAGGG)_2_) in pvT1 central region immediately adjacent the variable number of brown repeats (CTAGGG)_n_. |
| 17p (major)  Example sample: DER512 | The proximal region can vary in length between samples; but the fifth cyan (TTAGTG) repeat is replaced by a blue (GTAGTG) repeat so that the run of blue, green, black, black, cyan (GTAGTG-TTAGGG-CCAGAT-GCGAGG-TTAGTG) repeats present at the start of the pvT1 proximal region in other iciHHV-6B samples becomes blue, green, black, black, blue (GTAGTG-TTAGGG-CCAGAT-GCGAGG-GTAGTG) in the 17p (major) clade. |
| 19q  Example sample: COR264 | In the proximal region a brown repeat (CTAGGG) is replace by a dark green repeat (TTAGGG). This creates a run of three identical repeats (TTAGGG)_3_ that is shared by another iciHHV-6B sample (unknown integration site) and four acqHHV-6B samples.  N.B as with 11p, characterisation of pvT1 repeat patterns from other samples in the 19q-iciHHV-6B clade are needed to define the distinctive pvT1 features for this clade. |
