# Supplementary material for: Variation in human herpesvirus 6B telomeric integration, excision, and transmission between tissues and individuals
Source: eLife. 2021 Sep 21;10:e70452. doi: 10.7554/eLife.70452 (PMC8492063; doi:10.7554/eLife.70452)
Supplement: Supplementary file 4. [file elife-70452-supp4.docx]

**Supplementary File 4.** Measuring the percentage of acquired HHV-6B with a telomere, as an indicator of integration

|  | **SAL015** | **SAL027** | **SAL039** | **SAL040** | **SAL044** | **Tel-FA G1P2** | **K1** | **K10** |
| --- | --- | --- | --- | --- | --- | --- | --- | --- |
| Total DNA input (ng) | 3600 | 1800 | 1800 | 900 | 900 | 1800 | 225 | 225 |
| Cell equivalent | 5.45 x 10^5^ | 2.73 x 10^5^ | 2.73 x 10^5^ | 1.36 x 10^5^ | 1.36 x 10^5^ | 2.73 x 10^5^ | 3.41 x 10^4^ | 3.41 x 10^4^ |
| Reactions | 900 | 900 | 900 | 360 | 360 | 360 | 90 | 90 |
| Reactions with HHV-6B telomere | 5 | 3 | 9 | 2 | 19 | 4 | 0 | 4 |
| Copies of ciHHV-6 per cell | 9.17 x 10^-6^ | 1.1 x 10^-5^ | 3.30 x 10^-5^ | 1.47 x 10^-5^ | 1.39 x 10^-4^ | 1.47 x 10^-5^ | 0 | 1.17 x 10^-4^ |
| Copies of HHV-6B per cell | 0.0027 | 0.002933 | 0.001667 | 0.001333 | 0.012467 | 0.00087 | 0.000525 | 0.0124 |
| Percentage integrated /% | 0.34 | 0.38 | 1.98 | 1.1 | 1.12 | 1.69 | 0 | 0.95 |
