# Supplementary material for: Variation in human herpesvirus 6B telomeric integration, excision, and transmission between tissues and individuals
Source: eLife. 2021 Sep 21;10:e70452. doi: 10.7554/eLife.70452 (PMC8492063; doi:10.7554/eLife.70452)
Supplement: Supplementary file 5. [file elife-70452-supp5.docx]

**Supplementary File 5**. Variation between samples in the frequency of truncations at DR_L_-T2 and percentage lengthened.

| **Sample name** | **DNA source** | **Truncations at DRL-T2 per cell** | **Percentage lengthened** | **Lengthened per cell** |
| --- | --- | --- | --- | --- |
| NWA008 | Lymphoblasts | 0.0169 | 14.6 | 0.0025 |
| CEPH1375.02 | Lymphoblasts | 0.0091 | 4.55 | 0.0004 |
| COR264 | Lymphoblasts | 0.0078 | 15.8 | 0.0012 |
| 4B-11p15.5 | Lymphoblasts | 0.0248 | 3.33 | 0.0008 |
| 5B-17p13.3 | Lymphoblasts | 0.0158 | 0.00 | 0.00 |
| YOR546 | Lymphoblasts | 0.0136 | 18.2 | 0.0025 |
| 2B-9q34.3 | Lymphoblasts | 0.0172 | 0.00 | 0.00 |
| d37 | Pluripotent cells | 0.0093 | 80.9 | 0.0076 |
| CRL-1730 | Pluripotent cells | 0.019 | 37.0 | 0.007 |
| 401027 | Blood | 0.0025 | 63.7 | 0.0016 |
| 801018 | Blood | 0.0016 | 28.6 | 0.0005 |
| 211007 | Blood | 0.0025 | 77. 8 | 0.0019 |
| 704016 | Blood | 0.0009 | 100 | 0.0009 |
| 704021 | Blood | 0.0007 | 33.3 | 0.0002 |
| 801086 | Blood | 0.0032 | 68.8 | 0.0022 |
| 506007 | Blood | 0.0027 | 80.0 | 0.0022 |
| 607009 | Blood | 0.0084 | 67.6 | 0.0057 |
| 410005 | Blood | 0.0064 | 96.4 | 0.0061 |
| d32 | Sperm | 0.0063 | 4.41 | 0.0003 |
| d44 | Sperm | 0.0066 | 6.25 | 0.0004 |
| d56 | Sperm | 0.0059 | 8.87 | 0.0005 |
| Rx-F6a G4P2 | Saliva | 0.005 | 66.7 | 0.0033 |
| Rx-F6a G4P3 | Saliva | 0.0012 | 66.7 | 0.0008 |
