# Supplementary material for: Variation in human herpesvirus 6B telomeric integration, excision, and transmission between tissues and individuals
Source: eLife. 2021 Sep 21;10:e70452. doi: 10.7554/eLife.70452 (PMC8492063; doi:10.7554/eLife.70452)
Supplement: Supplementary file 6. [file elife-70452-supp6.docx]

**Supplementary File 6**. Measuring the frequency of truncations at DR_R_-T1 in various samples.

| **Sample name** | **DNA source** | **No. Cells (estimated from total DNA analysed)** | **No. of STELA reactions** | **No. of STELA products** | **No. of reactions with pvT1_R_** | **Estimated DR_R_-T1 telomeres per cell** |
| --- | --- | --- | --- | --- | --- | --- |
| NWA008 | Lymphoblasts | 4848 | 64 | 172 | 1 | 0.0002 |
| COR264 | Lymphoblasts | 4848 | 64 | 123 | 7 | 0.0014 |
| HGDP01065 | Lymphoblasts | 3864 | 64 | 61 | 11 | 0.0028 |
| 401027 | Whole blood | 8712 | 98 | 393 | 8 | 0.0009 |
| 211007 | Whole blood | 4848 | 64 | 143 | 3 | 0.0006 |
| 704021 | Whole blood | 2424 | 32 | 201 | 3 | 0.0012 |
| 506007 | Whole blood | 2424 | 32 | 200 | 7 | 0.0029 |
| d32 | Sperm | 4848 | 64 | 65 | 6 | 0.0012 |
| d56 | Sperm | 2424 | 32 | 70 | 4 | 0.0017 |
| Rx-F6a G2P2 | Saliva | 4848 | 64 | 132 | 4 | 0.0008 |
