# Supplementary material for: Variation in human herpesvirus 6B telomeric integration, excision, and transmission between tissues and individuals
Source: eLife. 2021 Sep 21;10:e70452. doi: 10.7554/eLife.70452 (PMC8492063; doi:10.7554/eLife.70452)
Supplement: Transparent reporting form [file elife-70452-transrepform1.pdf]

eLIFE
1st Floor
24 Hills Road
Cambridge CB2 1JP, UK
P 01223 855340
W elifesciences.org
T @elife

eLife's transparent reporting form

We encourage authors to provide detailed information *within their submission* to facilitate the interpretation and replication of experiments. Authors can upload supporting documentation to indicate the use of appropriate reporting guidelines for health-related research (see EQUATOR Network), life science research (see the BioSharing Information Resource), or the ARRIVE guidelines for reporting work involving animal research. Where applicable, authors should refer to any relevant reporting standards documents in this form.

If you have any questions, please consult our Journal Policies and/or contact us: editorial@elifesciences.org.

Sample-size estimation

- You should state whether an appropriate sample size was computed when the study was being designed
- You should state the statistical method of sample size computation and any required assumptions
- If no explicit power analysis was used, you should describe how you decided what sample (replicate) size (number) to use

Please outline where this information can be found within the submission (e.g., sections or figure legends), or explain why this information doesn't apply to your submission:

In general, sample size was not calculated during study design and the number of samples used in experiments was determined by the number of suitable samples available for analysis and amount of material available for each.
A spreadsheet containing information about all samples used has been included as a Supplementary Table 1.

Replicates

- You should report how often each experiment was performed
- You should include a definition of biological versus technical replication
- The data obtained should be provided and sufficient information should be provided to indicate the number of independent biological and/or technical replicates
- If you encountered any outliers, you should describe how these were handled
- Criteria for exclusion/inclusion of data should be clearly stated
- High-throughput sequence data should be uploaded before submission, with a private link for reviewers provided (these are available from both GEO and ArrayExpress)

Please outline where this information can be found within the submission (e.g., sections or figure legends), or explain why this information doesn't apply to your submission:

The information on the number of replicates performed for each assay is described in the Materials and Methods, in the figure legends and in accompanying supplementary tables.
Figure 1 and supplements: DRR-pvT1 PCR and sequencing was carried out once or the majority of samples but approximately 10 were repeated to confirm the sequence was the same.
Figure 4A: Saliva DNA was collected once and a single DNA extraction was used in three technical ddPCR replicates performed on different days. Figure 4C: Data from multiple STELA reactions were pooled to estimate the frequency of rare molecule (telomere) detection as shown in Supplementary Table 4. Figure 4E: three technical ddPCR replicates were performed at the same time for each saliva DNA sample.
Figure 5 and Supplement: (B) Data for each sample were pooled across 2-3 STELA blots and were not treated as technical replicates. (C and F) see Supplementary Table 5. (D) Trichostatin-A treated and untreated cells were cultured in triplicate and a separate DNA extracted was carried out for each treatment replicate (biological replicates). Two technical replicates of DRL-T2 truncation assay were carried out on each extracted DNA as describe in the material and methods and in figure 5 legend.
Figure 6: (B) Data from 2-3 STELA blots and corresponding secondary pvT1 gels (32 reactions of 500 pg per blot) were pooled to estimate the frequency of rare events (truncation) - data from different gels were not treated as replicates. See Supplementary table 6
We did not perform outlier analysis nor did we eliminate any data point on this criterion.

Statistical reporting

- Statistical analysis methods should be described and justified
- Raw data should be presented in figures whenever informative to do so (typically when N per group is less than 10)
- For each experiment, you should identify the statistical tests used, exact values of N, definitions of center, methods of multiple test correction, and dispersion and precision measures (e.g., mean, median, SD, SEM, confidence intervals; and, for the major substantive results, a measure of effect size (e.g., Pearson's r, Cohen's d)
- Report exact p-values wherever possible alongside the summary statistics and 95% confidence intervals. These should be reported for all key questions and not only when the p-value is less than 0.05.

Please outline where this information can be found within the submission (e.g., sections or figure legends), or explain why this information doesn't apply to your submission:

General information on data analysis given in the Methods section (Statistical Analysis). As required each figure legend has statistical information concerning the data representation, and details of tests. Data collected from individual samples are shown as individual points on graphs and presented in Supplementary Tables 2,4,5,6. Multiple correction was not carried out as only a small number of statistical tests were performed. The Mann-Whitney test was used to compare the mean ranks of independent groups in Figure 5C and 5F. This test was used because the data points within groups are independent, the groups are independent, and normal distributions were not assumed. The Wilcoxon test was used to compare treated and untreated samples (Figure 5D). Graphs show mean and standard error of the mean, and outlined in figure legends. Exact p-values are reported on graphs.

(For large datasets, or papers with a very large number of statistical tests, you may upload a single table file with tests, Ns, etc., with reference to sections in the manuscript.)

Group allocation

- Indicate how samples were allocated into experimental groups (in the case of clinical studies, please specify allocation to treatment method); if randomization was used, please also state if restricted randomization was applied
- Indicate if masking was used during group allocation, data collection and/or data analysis

Please outline where this information can be found within the submission (e.g., sections or figure legends), or explain why this information doesn't apply to your submission:

DNA samples were grouped based on the type of biological sample or cell-type (e.g. lymphoblastoid cell line, blood, saliva etc.). Masking was not used during data collection or analysis.

Additional data files ("source data")

- We encourage you to upload relevant additional data files, such as numerical data that are represented as a graph in a figure, or as a summary table
- Where provided, these should be in the most useful format, and they can be uploaded as "Source data" files linked to a main figure or table
- Include model definition files including the full list of parameters used
- Include code used for data analysis (e.g., R, MatLab)
- Avoid stating that data files are "available upon request"

Please indicate the figures or tables for which source data files have been provided:

Supplementary Table1.xls gives information about all the samples that were used for the study.

eLife Sciences Publications, Ltd is a limited liability non-profit non-stock corporation incorporated in the State of Delaware, USA, with company number 5030732, and is registered in the UK with company number FC030576 and branch number BR015634 at the address 1st Floor, 24 Hills Road, Cambridge CB2 1JP | August 2014
